# Gene Editing to Accelerate Crop Breeding

**DOI:** 10.3389/fpls.2022.889995

**Published:** 2022-05-27

**Authors:** Kanwarpal S. Dhugga

**Affiliations:** International Center for Maize and Wheat Improvement (CIMMYT), El Batan, Mexico

**Keywords:** accelerated breeding, backcrossing, disease resistance, gene editing, grain biofortification, maize lethal necrosis, rust resistance, SDN-1 -2 and -3

## Abstract

Recent advances in biotechnology have helped increase tissue transformation efficiency and the frequency and specificity of gene editing to an extent that introducing allelic variants directly in elite varieties has become possible. In comparison to the conventional approach of crossing an elite recipient line with an exotic donor parent to introduce the trait of interest followed by repeated backcrossing, direct introduction of major-effect allelic variants into elite varieties saves time and resources, and eliminates yield drag resulting from the residual donor genes at the end of backcrossing.

## Introduction

Forward breeding, which entails selection of recombinants with improved performance in appropriate environments, has been the driving force behind increasing crop yields over time. Discovery of heterosis, a term used to describe the improved performance of a hybrid as compared to its inbred parents, qualitatively increased the rate of yield improvement in the early phase of hybrid breeding (Sivasankar et al., 2012). Biotic and abiotic stresses drag yield down and contribute to the gap between the potential and the harvested grain yield (Duvick, 2005).

Indispensable as forward breeding is to crop improvement, particularly for complex traits and in stressful environments, it is a resource-intensive and time-consuming process. Even for simple traits inherited by single genes, several backcrosses (BC) are required to reconstitute the genome of the recipient parent. Another drawback of introgressing a trait through the conventional approach is the yield drag, a term used to refer to the reduction in grain yield from the unwanted genes from the donor parent that persist even after repeated backcrossing. Since these genes have not previously been subjected to selection for agronomic performance, they tend to reduce harvestable yield of the converted variety.

The number of genes from the donor parent that would still persist after *m* backcrosses, assuming no selection and no suppression of recombination, is n*d*(1/2)*^m^* where *d* is the fraction of loci that differ between the donor and the elite line and *n* is the total number of genes in the crop species. As an example, bread wheat has ∼110 K genes (Consortium et al., 2018). If a wild, donor accession differs from the recurrent parent at 30% of the loci, after four backcrosses more than a thousand genes from the donor parent would continue to be present in the converted variety.

In crosses between widely divergent lines, limited recombination could limit the proportion of the genomic segments of the donor parent that are introduced into the recurrent parent’s genome but could also pose a challenge in reducing the size of the introgressed donor segment, increasing the chances of linkage drag (Hao et al., 2020).

Markers could assist in reducing, but not eliminating, the donor parent genomic segments at BC1 stage. Breeding programs operating with limited resources would find it challenging to employ markers at this step. The choice, nevertheless, between introducing a gene variant into an elite variety without any accompanying donor genes using modern technology vs. forward breeding is obvious.

Speed breeding offers an alternative to reduce time in advancing generations in a controlled environment (Watson et al., 2018). It is not easily suited, however, for crops with large plants, like maize, pearl millet, and sorghum.

In the subsequent sections, I present the advantages gene editing has over conventional or speed breeding for at least the simply inherited traits.

## Gene Editing Can Reduce the Time to Product Development and Eliminate Yield Drag

Genetic engineering to introduce traits for which sufficient natural variation was not available proved to be effective in combating insect pests and weeds (Rafalski, 2017). However, the benefits of the GM crops have mostly been realized by the farmers of the developed countries (Klümper and Qaim, 2014). The cost of the seed and consumer resistance against the GM crops have kept them out of the developing countries, particularly Africa (Rafalski, 2017).

Modern technologies have made it possible to accelerate improvement of genetically simple traits, which are controlled or influenced by single or a few genes, without the concerns associated with the GM crops.

The field of gene editing has progressed through several phases starting with oligo-mediated editing in the 1980s (Carroll, 2017). The main hurdle in its adoption was the low frequency of the edited events (Zhu et al., 1999, 2000). A relatively new technique, clustered regularly interspersed short palindromic repeats (CRISPR) and CRISPR-associated protein 9 (Cas9), referred to as CRISPR-Cas9, has revolutionized the field of gene editing because of its ease of use, specificity, and a high success rate (Svitashev et al., 2015).

CRISPR-Cas9-mediated gene editing has been used to mutate genes either through the spontaneous non-homologous end-joining (NHEJ) after the double-strand break at the precise site targeted by the guide-RNA or through gene deletion by using two guide-RNA molecules simultaneously. This has been referred to as site-directed nuclease scenario-1 (SDN1) (Podevin et al., 2013; Savitashev et al., 2015). The other two scenarios, SDN2 and SDN3, entail template-mediated nucleotide changes and insertion of a gene or a DNA fragment into the genome, respectively.

Myriad examples of gene editing using the CRISPR-Cas system in crop plants are listed in recent reviews (Schenke and Cai, 2020; Tiwari et al., 2021). Most of what I discuss in the subsequent sections is related to accelerating varietal improvement by directly introducing high-value traits into elite lines (Gao et al., 2020).

To demonstrate the effect of a gene variant via gene editing, experimental lines, which are older accessions, have been used in a great majority of published reports in crop plants, obviously because it is difficult to transform elite varieties directly (Schenke and Cai, 2020; Tiwari et al., 2021). To transfer the newly created trait from an experimental line into elite varieties requires crossing and backcrossing, which negates the advantage of gene editing with regard to shortening the time to product development as well as in eliminating yield drag.

**FIGURE 1:**
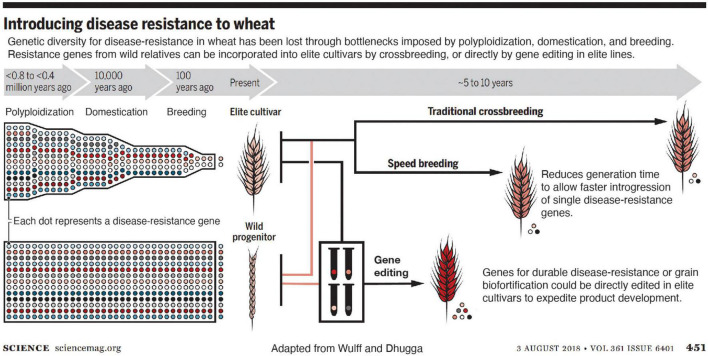
Introducing disease resistance into wheat. Genetic diversity for disease resistance in wheat has been lost through bottlenecks imposed by polyploidization, domestication, and breeding. Resistance genes from wild relatives can be incorporated into elite cultivars by crossbreeding, which is sped up by speed breeding, and further accelerated by gene editing directly in elite lines (from Wulff and Dhugga, 2018). Reprinted with permission from AAAS.

Editing a gene directly in elite varieties eliminates the need for backcrossing (Figure 1). After self-pollinating or outcrossing the edited plants to the non-edited plants of the same genetic makeup accompanied by simultaneous screening for any unintended changes in the genome with highly sensitive molecular tools ensures no elements of the vector backbone remain in the edited plants (Zastrow-Hayes et al., 2015). According to my colleagues in industry, commercialization of the same trait advanced through conventional breeding as generated by gene editing reduced the time to market the improved variety by approximately two-thirds in the latter case (Figure 1). Savings in field resources, which constitute one of the most expensive components of varietal development, is proportional to the time saved; only 2–3 instead of 5–6 generations are needed for the gene edited plants to commercialize as compared to forward breeding.

## Bottlenecks in Gene Editing in Elite Varieties Are Being Overcome

The hurdle of transforming elite varieties directly has recently been overcome by including cell morphogenesis genes in the transformation vector (Lowe et al., 2016, 2018a,2018b; Debernardi et al., 2020). In maize, inclusion of cell morphogenesis genes in the transformation vector and finetuning their expression made it possible to transform elite varieties directly with very high efficiency (Lowe et al., 2016, 2018a). Similarly, transformation efficiency was significantly improved in wheat by including a growth regulating factor and its cofactor in the transformation vector (Debernardi et al., 2020). A previously published and most commonly used protocol worked inconsistently in different laboratories (Ishida et al., 2015). The recent advances in high efficiency transformation have paved the way to edit genes directly in elite lines, at least in major crops.

Under a partnership with Corteva Agriscience, we at CIMMYT have successfully transformed elite lines of tropical maize with nearly perfect efficiency. This has opened the door to use gene editing approaches directly in the commercial lines.

## No Dearth of Traits in Crop Plants That Can Be Improved by Gene Editing

Disease resistance and grain biofortification are two of the areas where gene editing can help expedite crop improvement. Some other traits specific to different crops are also amenable to improvement by gene editing.

Rancidity in pearl millet flour, which is caused by oxidation of unsaturated fatty acids released by lipases, limits its shelf life (Goyal and Chugh, 2017). It could potentially be reduced by addressing a few qualitative steps in fatty acid formation and triglyceride hydrolysis. Suppressing a *fatty acid desaturase* (*FAD2*), which converts oleic to linoleic acid, specifically in the seed substantially increased the proportion of oleic acid in the oil (Damude and Kinney, 2008). As mutants in the *FAD2* had pleiotropic effects, this gene might prove to be a challenge in reducing rancidity through a knockout in pearl millet. An alternative could be to replace the native promoter of *FAD2* with the one that is not expressed, or is expressed at a low level, in the seed but is expressed normally in other tissues (Shi et al., 2017). Lipases, which release fatty acids from triglycerides upon grinding of the grains into flour, offer an alternative target to reduce rancidity in pearl millet (Goyal and Chugh, 2017).

Striga is an obligate root parasitic weed that affects maize and sorghum production in semiarid tropics in Asia and Africa, particularly in nitrogen-poor soils. Its seeds germinate only when they sense a signal secreted by the host roots (Gobena et al., 2017). Mutation in a single gene involved in the formation of a specific type of strigolactone significantly improved Striga resistance in sorghum (Gobena et al., 2017). Instead of backcrossing this mutant gene into other elite varieties, it could be directly created by gene editing.

Rusts affect wheat crop more than any other disease. Approximately one-fifth of the wheat crop is lost to diseases every year (Oerke, 2006). Resistance against fungal diseases in wheat like powdery mildew and rusts can be significantly improved by editing single or a few genes. Once disease resistance breaks down, new genes for resistance must be introduced. The source of disease resistance genes is generally found in wild or genetically divergent accessions (Wulff and Dhugga, 2018).

Host resistance, which is attributed to resistant (R) genes, results from a hypersensitive response of the host, which kills the cells around the infected cell and thus limits the spread of the pathogen (Gill et al., 2015). These genes are generally involved in cell signaling. This type of resistance tends to break down with time, however, requiring the introduction of new sources of resistance, again necessitating backcrossing to the recipient line. Non-host resistance, in contrast, involves metabolic or transport proteins. It allows the pathogen to grow at a slow rate but without significantly affecting grain yield. It is also referred to as durable resistance or adult plant resistance (APR). Further, the APR genes confer resistance against a broad spectrum of fungal pathogens (Moore et al., 2015). Because of its durability, CIMMYT breeders prefer APR to host resistance and have integrated it into their breeding program.

Three APR loci, Lr34, Lr46, and Lr67 are known in wheat and genes for two (Lr34 and Lr67) have been isolated. Whereas Lr34 encodes an ATP-binding cassette (ABC) transporter, Lr67 encodes a hexose transporter (Krattinger et al., 2009; Moore et al., 2015). Just to highlight the durability of resistance conferred by these genes, Lr34 has not broken down for over the 100 years it has been available to the breeders and thus farmers (Moore et al., 2015). In both Lr34 and Lr67, mutations occur in the transmembrane domains of the respective proteins they encode, apparently making the proteins non-functional. Loss of function of the mutated protein has been demonstrated for Lr67 in a heterologous system but not for the Lr34 protein, apparently because its substrate is not known (Krattinger et al., 2009; Moore et al., 2015). ABC family of transporters facilitates the transport of a wide variety of substrates and is also referred to as multidrug resistance (MDR) protein family in bacteria. However, like Lr67, the causal mutations occur in two of the transmembrane domains in Lr34 as well, one of which, a tyrosine to histidine change, is expected to attenuate, if not destroy, its function.

These non-functional transporters, although might normally be involved in the transport of plant metabolites, likely confer resistance against the fungal pathogens by blocking the transport of toxins or effectors that kill the plant cells. The mutations are partially dominant, which can be explained by the dimerization of the encoded proteins. Assuming the mutant and the wildtype alleles express at the same level in a heterozygote, three-fourths of the dimers would be expected to be defective. Many transporters are known to function as dimers (Feng and Frommer, 2015).

At least for Lr67, as the mutant protein that confers resistance is non-functional, an exact replication of the mutation in the elite lines is not necessary. Simple inactivation via an SDN1 knockout should phenocopy the spontaneous mutant (Moore et al., 2015). Same logic could be applied to Lr34.

Although homeoalleles for each of the isolated APR genes are present, genetic alteration of a single homeolog confers resistance (Krattinger et al., 2009; Moore et al., 2015). With gene editing, it is possible to knockout all three homeoalleles and then test them individually and in combinations to determine whether they further augment resistance. If so, it will help expand the repertory of the tools to improve disease resistance. Since the mutations will be in the same exact genetic background, constituting these combinations and testing them for rust resistance would be straightforward.

Another target for SDN1 editing in wheat is resistance against powdery mildew, which is controlled by Mildew Locus O (MLO), a dominant suppressor of resistance. All three homeoalleles must be knocked out to confer resistance (Acevedo-Garcia et al., 2017; Li et al., 2022). Li et al. (2022) in fact succeeded in generating mutations in all three loci in elite lines directly, a feat that can be reproduced in other susceptible commercial varieties.

Resistance against Fusarium head blight in wheat could be directly introduced into elite varieties by knocking out a histidine-rich calcium-binding protein (Su et al., 2019).

**FIGURE 2:**
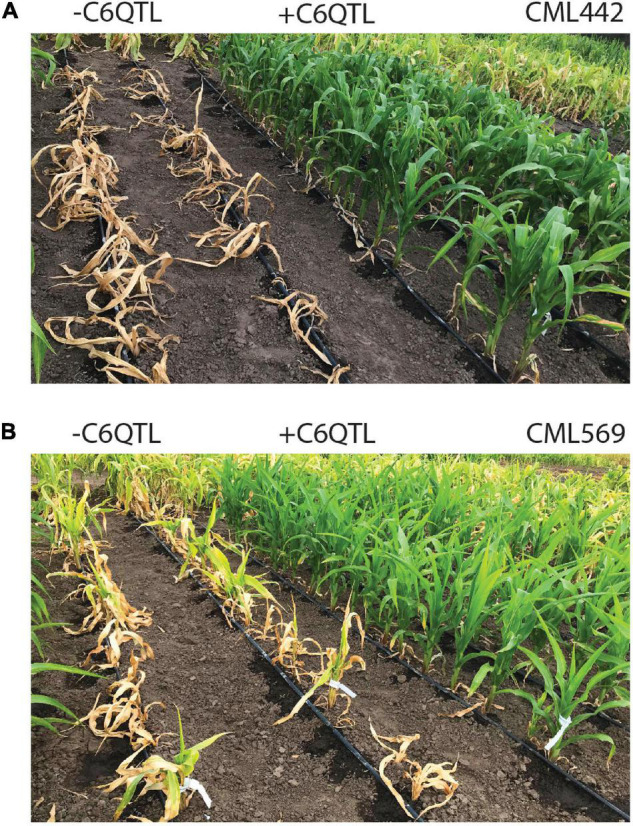
A large-effect QTL provides strong protection against maize lethal necrosis (MLN). Inbred line CML442 **(A)** and CML569 **(B)** after inoculation with the MLN viruses without (left) or with (right) the C6QTL from the inbred donor line KS23-6 after four backcrosses to the recurrent parent. The lines were screened for MLN in Naivasha, Kenya. Picture credit: Michael Olsen.

Maize lethal necrosis (MLN), a viral disease that swept through Kenya starting a decade ago and spread to the neighboring countries, devastated crop production (Boddupalli et al., 2020). CIMMYT identified a strong QTL that provides qualitative resistance against MLN (Murithi et al., 2021). The QTL, which was completely recessive, was present in an exotic line, which is unrelated to the African germplasm. The strength of this QTL is displayed in Figure 2 where the same inbred line with or without the QTL was inoculated with MLN. As forward breeding to introgress this QTL continues, we have also fine mapped it and are in the process of identifying the causal gene through gene editing. Once identified, this gene could be directly knocked out in the susceptible elite lines, which could be turned around in 2–3 years instead of 7–8 years it takes to introgress with backcrossing.

Editing herbicide tolerance into varieties directly could help reduce drudgery for women in Africa where they have to manually remove weeds from the crops (Savitashev et al., 2015; Sun et al., 2016). Affordability of herbicides by the smallholder farmers remains a question mark, however, so the penetration of this trait remains uncertain.

Grain biofortification is critical for proper development of the children in developing countries where a lack of micronutrients and vitamins can cause developmental defects (Wen et al., 2022). Phytic acid chelates divalent cations and keeps them from being absorbed in the digestive system. A twofold variation of grain phytic acid in a breeding population suggested it was possible to improve divalent cation availability within the existing range of genetic variability (Wen et al., 2022). Shukla et al. (2009) demonstrated that it was possible to reduce grain phytic acid in maize by gene editing, targeting the enzyme that phosphorylates inositol. The same approach could be used to reduce phytic acid in already released commercial varieties, particularly targeted for increased iron and zinc contents (Wen et al., 2022). Similarly, provitamin-A in the grains of maize and other cereals could be improved by knocking out the genes that divert the substrate to other reactions as well as the ones that oxidize beta-carotene (Sestili et al., 2019).

Dough from wheat flour turns dark because of polyphenol oxidase (PPO) activity. Similarly, peeled potatoes turn brown if left exposed to air. Gene editing has been used to knock out a PPO gene in potato, which reduced browning (González et al., 2020). We are using a similar approach in wheat to prolong dough longevity.

## Prospects of Gene Editing in Crop Improvement: SDN1, SDN2, or SDN3

Homology directed repair (SDN2), promoter swapping, and allele replacement or insertion (SDN3) have been successfully demonstrated in crop plants (Savitashev et al., 2015; Shi et al., 2017). Low frequency of the edited events, which would be lower still in crops where transformation is a challenge, and extensive screening required to identify the targeted changes would limit the use of these approaches to high-value traits, however (Zhu et al., 1999, 2000; Shukla et al., 2009). An example of the large-effect, high-value QTL is displayed in Figure 2. These were the hurdles that kept the prior gene editing technologies from wide adoption (Zhu et al., 1999, 2000; Shukla et al., 2009; Carroll, 2017). Further, SDN3-derived events would invite increased regulatory scrutiny (Podevin et al., 2013). As has been the case thus far, gene editing via SDN1 would most likely continue to dominate trait improvement in commercial germplasm followed by limited use of SDN2 and SDN3 in that order for the traits that justify investment of additional resources.

Editing of genes with major effect directly in elite varieties, mostly with SDN1, is already underway and will help expedite crop improvement going forward. There is no dearth of high-value traits controlled by single genes the desired variants of which can be reproduced using SDN1, thus eliminating the pleiotropic effects associated with the residual donor genes that cannot be completely removed by conventional plant breeding.

## Data Availability Statement

The original contributions presented in the study are included in the article further inquiries can be directed to the corresponding author.

## Author Contributions

The author confirms being the sole contributor of this work and has approved it for publication.

## Conflict of Interest

The author declares that the research was conducted in the absence of any commercial or financial relationships that could be construed as a potential conflict of interest.

## Publisher’s Note

All claims expressed in this article are solely those of the authors and do not necessarily represent those of their affiliated organizations, or those of the publisher, the editors and the reviewers. Any product that may be evaluated in this article, or claim that may be made by its manufacturer, is not guaranteed or endorsed by the publisher.
